# Genetic Variants in Smoking-Related Genes in Two Smoking Cessation Programs: A Cross-Sectional Study

**DOI:** 10.3390/ijerph18126597

**Published:** 2021-06-19

**Authors:** Gloria Pérez-Rubio, Luis Alberto López-Flores, Ana Paula Cupertino, Francisco Cartujano-Barrera, Luz Myriam Reynales-Shigematsu, Mariana Ramírez, Edward F. Ellerbeck, Rosibel Rodríguez-Bolaños, Ramcés Falfan-Valencia

**Affiliations:** 1 HLA Laboratory, Instituto Nacional de Enfermedades Respiratorias Ismael Cosío Villegas, Calzada de Tlalpan 4502, Sección XVI, Mexico City 14080, Mexico; glofos@yahoo.com.mx (G.P.-R.); llopezf92@gmail.com (L.A.L.-F.); 2 Department of Cancer Prevention and Control, Hackensack University Medical Center, Hackensack, NJ 07601, USA; paula.cupertino@hackensackmeridian.org (A.P.C.); francisco.cartujano@hackensackmeridian.org (F.C.-B.); 3 Department of Tobacco Research, Instituto Nacional de Salud Pública, Cuernavaca 62100, Mexico; lreynales@insp.mx (L.M.R.-S.); rrodriguez@insp.mx (R.R.-B.); 4 Department of Preventive Medicine and Public Health, University of Kansas Medical Center, Kansas City, KS 66160, USA; mramirez3@kumc.edu (M.R.); eellerbe@kumc.edu (E.F.E.)

**Keywords:** nicotine addiction, smoking cessation, *CHRNA3*, *CHRNA5*, *NRXN1*, *DRD4*, *HTR2A*, *CYP2A6*, e-Decídete

## Abstract

Previous studies have identified variants in genes encoding proteins associated with the degree of addiction, smoking onset, and cessation. We aimed to describe thirty-one single nucleotide polymorphisms (SNPs) in seven candidate genomic regions spanning six genes associated with tobacco-smoking in a cross-sectional study from two different interventions for quitting smoking: (1) thirty-eight smokers were recruited via multimedia to participate in e-Decídete! program (e-Dec) and (2) ninety-four attended an institutional smoking cessation program on-site. SNPs genotyping was done by real-time PCR using TaqMan probes. The analysis of alleles and genotypes was carried out using the EpiInfo v7. on-site subjects had more years smoking and tobacco index than e-Dec smokers (*p* < 0.05, both); in *CYP2A6* we found differences in the rs28399433 (*p* < 0.01), the e-Dec group had a higher frequency of TT genotype (0.78 vs. 0.35), and TG genotype frequency was higher in the on-site group (0.63 vs. 0.18), same as GG genotype (0.03 vs. 0.02). Moreover, three SNPs in *NRXN1*, two in *CHRNA3*, and two in *CHRNA5* had differences in genotype frequencies (*p* < 0.01). Cigarettes per day were different (*p* < 0.05) in the metabolizer classification by *CYP2A6* alleles. In conclusion, subjects attending a mobile smoking cessation intervention smoked fewer cigarettes per day, by fewer years, and by fewer cumulative pack-years. There were differences in the genotype frequencies of SNPs in genes related to nicotine metabolism and nicotine dependence. Slow metabolizers smoked more cigarettes per day than intermediate and normal metabolizers.

## 1. Introduction

Cigarette consumption is a global public health problem. In Mexico, around 60,000 people die a year (between 165 and 180 deaths a day) due to diseases associated with tobacco use, according to the statistics from the General Directorate of Epidemiology of the Ministry of Health. According to the latest National Survey on Drug, Alcohol, and Tobacco Consumption (ENCODAT) 2016–2017 [1], there are more than 15.6 million smokers in Mexico, and the most vulnerable group is young people between 12 and 15 years of age. Both men and women smoke tobacco alike [1]. In addition, the Global Adult Tobacco Survey 2015 (GATS) reports that 17.6% of the population between 12–65 years old smoke tobacco; of these, 73.6% are interested in quitting smoking, 56.1% have tried to quit such drug, but only 1.2% underwent some treatment to leave; only 3.5% used pharmacotherapy, 5.9% used counseling, and 90.6% used willpower [2]. Furthermore, it is unclear whether subjects with different smoking behavior characteristics can access mobile smoking cessation services. Tobacco smoking is a common and multifactorial disease where the environmental and genetic factors participate in a complex network where gene products are involved in addictive pathways [3]. Genome-wide association studies (GWAS) have identified some *loci* with smoking patterns of cigarette consumption in nicotinic cholinergic receptors genes (*CHRNA5*-*CHRNA3*-*CHRNB4*) [4], neurotransmitter receptors’ genes (*HTR2A*, *DRD4*, etc.), as well as those involved in the metabolism of nicotine such as *CYP2A6*, which is a liver enzyme that metabolizes between 70%–80% of the nicotine that enters the body in cotinine [5]. At the genetic level, it has been reported that there are more than 30 allelic variants that affect the enzymatic activity [6,7]. The CYP2A6*1A allele is considered as the reference, while the presence of CYP2A6*1B causes low expression of the mRNA, in vitro studies have shown a low rate of nicotine metabolism [8]. Several alleles have been described as decreased enzymatic function, and mainly these alleles are produced by SNPs (CYP2A6*5, *7, *9, *18, *21, *23 *25, *28, and *35 [9]). Several GWAS performed in Asian, European, and African populations identified polymorphisms in the *CHRNA5* and *CYP2A6* genes strongly associated with the risk of smoking [10], being a heavy smoker (≥20 cigarettes per day) [11], to a better success in the treatment to stop smoking [9] or in the development of diseases caused by cigarette smoking (mainly COPD and lung cancer) [12,13].

We aimed to describe smoking behavior, allele, and genotype frequencies of polymorphic variants in six candidate genes in a population of smokers recruited by a mobile smoking cessation intervention and compare to people attending an institutional smoking-cessation program.

## 2. Materials and Methods

### 2.1. Population Study

A cross-sectional study was performed with baseline data from two different interventions for quitting smoking. (1) e-Decídete! program daily smokers and (2) daily smokers attending an institutional smoking cessation program on-site were included.

e-Decídete! program: this study aimed to assess the feasibility and acceptability of an innovative, personalized, and interactive smoking cessation mobile intervention developed for Mexican smokers. Participants were recruited through printed posters and multimedia venues, including ads through the National Institute of Public Health (Instituto Nacional de Salud Pública, Morelos, Mexico) website, Facebook, and local radio announcements. Potential participants emailed or called study personnel to learn more about the study. An eligibility assessment was conducted over the phone. Eligible participants were of Mexican origin, ≥18 years of age, had smoked for at least 6 months, smoked at least 3 days per week, interested in quitting within the next 30 days, had a cellphone with text messaging capacity, and were willing to complete baseline and 12-week follow-up surveys. Participants were excluded from the study if they were planning to move within the next six months, consumed other forms of tobacco (including e-cigarettes), or had another household member enrolled in the study. All subjects gave informed consent prior to participation in the study. Participants received 300 MXN (≈15 USD) at baseline and follow-up as an incentive for their time and transportation. This study was conducted between March and August 2017 at the Medical Center of the Autonomous University of the State of Morelos, located in Cuernavaca, Morelos, Mexico.

Smokers attending smoking cessation program on-site: participants were recruited from a quit-smoking help clinic from the Department of Research in Smoking and COPD of the National Institute of Respiratory Diseases Ismael Cosío Villegas (Instituto Nacional de Enfermedades Respiratorias Ismael Cosío Villegas (INER)) of Mexico. Only Mexican Mestizos by ancestry (Mexican-birth parents and grandparents) were included. Individuals with bronchial asthma, COPD, bronchiectasis, active tuberculosis, lung cancer, cystic fibrosis, hypersensitivity pneumonitis, or idiopathic pulmonary fibrosis were excluded from the study. To reduce difference among main covariates and avoid confounding factors, we matched intentionally this sample group by age and cigarettes per day.

The Human Subjects Committees of the Mexican National Institute of Public Health (INSP) and the Respiratory Diseases National Institute Ismael Cosio Villegas (INER, protocol numbers B07-17 and B15-16) approved the study procedures. The participants were invited to participate in the present research study and were informed about its aim. The participants then signed a letter of informed consent and were provided with an assurance-of-personal-data document. The Research Institute’s Committee approved both documents of Science and Bioethical Research. Each participant was assigned an alphanumeric key to assure confidentiality.

### 2.2. Samples Obtention

An 8-mL volume of peripheral blood was obtained using venipuncture techniques and collected in a tube with EDTA as an anticoagulant for subsequent DNA extraction using a BDtract DNA Isolation Kit (Maxim Biotech, San Francisco, CA, USA). The DNA was quantified by ultraviolet absorption at a 260-nm wavelength using a NanoDrop instrument (Thermo Scientific, Waltham, MA, USA). Contamination with organic compounds and proteins was determined by establishing the relationship between the 260/240 and 260/280 readings, respectively. The samples were considered free of contaminants when the relationship was found to be between 1.7 and 2.0.

### 2.3. Single Nucleotide Variation Selection

Two types of polymorphisms were evaluated: those SNPs forming CYP2A6 alleles involved in enzymatic activity alterations and a set of polymorphisms previously found associated with smoking and/or its behavior in a Mexican mestizo population.

SNPs in *CYP2A6* were selected according to their allele assignation by the Pharmacogene Variation Consortium [14]. Minor alleles from these SNPs could be in more than an allele, whereby we select a specific set of SNPs that could discriminate a particular allele; therefore, each SNP (or set of SNPs) corresponds to a single allele. Fourteen polymorphisms at the *CYP2A6* gene were included, of which 10 are missense, 2 synonymous, one 2KB upstream, and one intron variant, spanning 6647 bp at q13.2 in chromosome 19. Next, we search for the enzyme’s main effect by carrying each allele in each subject genotype and then classify it as a type of normal, intermediate, or slow metabolizer. Table 1 collects information about molecular characteristics and frequencies for SNPs in *CYP2A6* related to the enzymatic activity.

Moreover, a set of seventeen polymorphisms previously reported on the fine mapping of six *loci* were evaluated; these include 12 intron variants, 1 missense (*CHRNA5*: rs16969968, [Asp398Asn]), two 2KB-upstream, one synonymous (*HTR2A*: rs6313, [Ser34Ser]) and one intergenic variant. These 17 SNPs were selected because they were associated with cigarette smoking or a high degree of nicotine addiction in Mexican mestizo smokers. [15,16]. Characteristics of evaluated SNPs are disposable in Table 2.

### 2.4. Genotyping

Genotyping was performed using TaqMan allelic discrimination real-time PCR with pre-designed probes and a real-time PCR thermocycler (7300 Real-Time PCR Systems, Applied Biosystems/Thermo Fisher Scientific Inc., Singapore); considering the allelic discrimination and confirmed by absolute quantitation; three controls without template (contamination controls) were included for each genotyping plate, and 5% of the samples included in the study were genotyped in duplicate as controls for allele assignment. Data interpretation was conducted using Sequence Detection Software (SDS v. 1.4, Applied Biosystems, Waltham, MA, USA). VIC and FAM fluorophores were used for alleles A and B, respectively.

### 2.5. Statistical Analysis

The software SPSS v.20.0 was used (SPSS software, IBM, New York, NY, USA) to determine each variable’s median and interquartile range. For the allele and genotype frequencies were employed Epidat version 3.1 software. [17]. For comparisons between genotype frequencies, Pearson’s χ^2^ test was employed when they had individuals in three genotypes in one group and at least two genotypes in the other. Otherwise, we use the Fisher exact test when both groups had only two genotypes each.

## 3. Results

The *Vive sin Tabaco…¡Decídete!*: Single-Arm Pilot Study is a mobile smoking cessation intervention previously described [18].

### 3.1. Study Population

Thirty-eight smokers attending the e-Decídete! program (e-Dec) were included, while smokers attending an institutional smoking cessation program (on-site) were ninety-four. The demographic variables for both study groups are presented in Table 3. This shows the median and minimum, and maximum values in each variable.

### 3.2. Genotype Frequencies

Of the 15 SNPs located in *CYP2A6*, only 6 (rs28399433, rs28399434, rs8192720, rs1137115, rs2431413, and rs5031017) were polymorphic in at least two genotypes in a group. Comparing genotype frequencies between both groups, we find differences in the rs28399433 (*p* < 0.0001), in the e-Dec group there was a higher frequency of TT genotype (0.78 vs. 0.35), and TG genotype frequency was higher in the on-site group (0.63 vs. 0.18), same as GG genotype (0.03 vs. 0.02). Moreover, in rs2431413, genotype frequencies between both groups were different, in which only the on-site group had variation in frequency. Otherwise, rs1137115 (*p* = 0.768) and rs5031017 (*p* = 0.700) had similar genotype frequencies with non-significant statistical *p*-values; these data are shown in Table 4. Due to the lack of minor allele homozygous genotype individuals, we compare frequencies of major allele homozygous versus heterozygous genotypes in rs28399434 (*p* = 0.181) and rs8192720 (*p* = 0.287), with no statistically significant differences.

We evaluated seventeen SNPs in five genes (*NRXN1*, *DRD4*, *HTR2A*, *CHRNA3*, *CHRNA5*, and near to *CYP2A6*) related to nicotine addiction; data are shown in Table 5. In *NRXN1* there were differences in three SNPs, rs10189159 (*p* = 0.0004) had higher genotype frequency TT in e-Dec group (0.84 vs. 0.46); instead, TC (0.47 vs. 0.13) and CC (0.05 vs. 0.02) genotype frequencies were higher in the on-site group; the rs985919 (*p* = 0.0333) had higher genotype frequency TT in e-Dec group (0.55 vs. 0.30) but genotype (TG) had minor frequency (0.36 vs. 0.57) and same for genotype GG (0.09 vs. 0.29). The rs1882296 had statistical significance differences (*p* = 0.0348), the AA genotype had a higher frequency in the e-Dec group (0.68 vs. 0.47) and the genotypes AG and GG had higher frequencies in on-site group (0.45 vs. 0.28 and 0.06 vs. 0.02, respectively). Two SNPs in *CHRNA3* had differences statistically significant in genotype frequencies, rs1317286 (*p* = 0.0311) AA genotype frequency was higher in e-Dec group (0.73 vs. 0.52) and heterozygous AG genotype was higher in the on-site group (0.42 vs. 0.23), minor allele GG had the same tendency (0.05 vs. 0.02); rs615470 (*p* = 0.0355) had differences among genotype frequencies in groups where TT genotype frequency was higher in the e-Dec group (0.81 vs. 0.60) and both TC (0.35 vs. 0.15) and CC (0.04 vs. 0.02) genotypes were higher in the on-site group. Two SNPs in *CHRNA5* had statistically significant differences in genotype frequencies, rs17408276 (*p* = 0.0161) TT genotype frequency was higher in e-Dec group (0.81 vs. 0.56) and the heterozygous TC genotype was higher in the on-site group (0.41 vs. 0.15), minor allele CC genotype was similar in both groups (0.02 vs. 0.02); rs951266 (*p* < 0.0001) had differences among genotype frequencies in groups where CC genotype frequency was higher in e-Dec group (0.94 vs. 0.54) and both CT (0.40 vs. 0.05) and TT (0.05 vs. 0.00) genotypes were higher in on-site group.

### 3.3. Metabolizer Classification by CYP2A6 Alleles

We classify individuals by metabolizer type according to the polymorphic SNPs in our sample (rs28399433, rs28399434, rs8192720, rs2431413, and rs5031017) by their effect reported in the literature. We determine that two or more minor alleles of these SNPs were a slow metabolizer, one minor allele was an intermediate metabolizer and none-minor alleles were a normal metabolizer. We excluded rs1137115 because it was a non-specific marker of decreased function alleles. With this classification we could evaluate normal metabolizers (*n* = 19), intermediate metabolizers (*n* = 39), and slow metabolizers (*n* = 48). Comparing tobacco-related variables in these groups, only cigarettes per day were different in this classification (*p* = 0.038), in which slow metabolizers were those who smoked slightly more (10 (9.5–15)) than intermediate (10 (8–14)) and normal (8 (5–11)) metabolizers.

**Figure 1:**
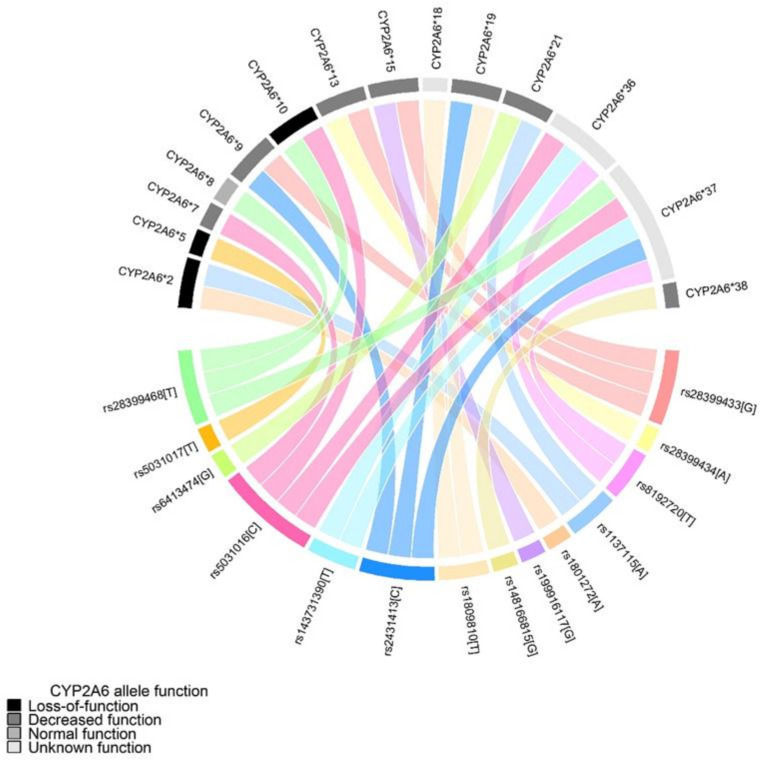
Circle plot for *CYP2A6* alleles. *SNPs assign CYP2A6 alleles and functions* according to PharmVar Consortium.

Figure 1 shows how different *CYP2A6* alleles can harbor more than one SNP from the selected panel. Subjects were categorized into groups by their *CYP2A6* allele effect reported in the literature. *CYP2A6* were grouped as loss-of-function (*2, *5, and *10), decreased (*7, *9, *13, *15, *19, *21, and *38) and normal function (*1, *8), the remaining alleles did not have information in the literature (*18, *36, and *37). Once each subject had genotyped their corresponding alleles, their genotypes were grouped into the enzymatic activity by nicotine metabolite ratio, which is evaluated by the 3HC/COT ratio [19] as normal (two normal function alleles), intermediate (one normal function allele and one decreased function allele) or slow metabolizers (one normal function allele and one loss-of-function allele; one decreased function allele and loss-of-function allele; two decreased function alleles; two loss-of-function alleles).

### 3.4. Matched Sub-Analysis

We matched e-Dec with on-site groups by age and gender to avoid confounding variables due to group differences in demographic variables. We keep the thirty-eight e-Dec and sixty-two on-site participants. Although these groups have similar age (*p* = 0.847) and gender proportions (*p* = 0.542), both groups have differences in tobacco smoking variables. e-Dec group smoked fewer cigarettes per day (*p* = 0.043), by fewer years (*p* = 0.014), and by fewer cumulative pack-years (*p* = 0.003). Interestingly, we obtained a marginal difference in the Fagerström Test for Nicotine Dependence (FTND) (*p* = 0.053) with higher values in the e-Dec group. In the rs28399433 genotype evaluation, this kept with differences among both groups (*p* < 0.0001), mainly in TT (0.78 vs. 0.29) and TG (0.18 vs. 0.67) genotype frequencies. The remaining SNPs lack statistically significant differences among their genotype frequencies.

## 4. Discussion

In the current study, we report for the first time *CYP2A6* SNPs in Mexican mestizo smokers and their distribution among two different smoking cessation programs. Previously, we had reported other SNPs in smoking-related genes as *NRXN1*, *DRD4*, *HTR2A*, *CHRNA3*, *CHRNA5*, and *CYP2A6* in Mexican mestizo, focused on smokers enrolled in a smoking cessation program, mostly with high tobacco consumption and nicotine dependence [15,16,20]. Here, we describe genotype frequencies of SNPs in smoking-related genes on an innovative based in text messages program, which enrolled smokers from the general population.

In our study groups, tobacco-related variables (cigarettes per day, years of smoking, and pack-years) do not reflect nicotine dependence assessed by FTND; despite individuals in the e-Dec group smoking less, they had a higher score in the questionnaire. Although the FTND application has been discussed in the Mexican Mestizo population [21], it is the most-employed test to assess nicotine dependence in the scientific literature.

Despite their effect on the enzyme activity, selected *CYP2A6* SNPs, according to assignation by the Pharmacogene Variation Consortium [14], only six were polymorphic; that is, no minor allele was identified in nine SNPs potentially related to alteration in the ability to metabolize nicotine. This is probably due to miscegenation in the Mexican mestizo population in which the ancestral Amerindian component reaches 55% [22], and SNPs reported as *CYP2A6* activity-related are mainly evaluated in Caucasian populations. We found differences among two cessation programs in two SNPs, rs28399433 and rs2431413; previously, the rs28399433 showed reduced apparent oral efavirenz clearance [23]; regarding nicotine metabolism, women and normal nicotine metabolizers (harboring the common allele) may benefit more from varenicline over nicotine replacement therapy [24]. Previously, our workgroup identified an association with a younger age at onset smoking [16], smoking status, age of onset, and psychological dependence in Mexican mestizo families [15,25].

With regard to single nucleotide variants analyzed in the genes related to those involved in the dopaminergic and serotonergic pathways, we identified differences in the genotype frequency among both groups in three SNPs in *NRXN1* gene, the rs10189159, rs985919, and rs1882296; the latter revealed in an in silico analysis that rs1882296/T had a high level of homology with Hsa-miR-6740-5p, which encodes a putative miRNA that targets glutamate receptor subunits (GRIA2, GRID2) and GABA receptor subunits (GABRG1, GABRA4, GABRB2). In contrast, rs1882296/C had a high homology level with Hsa-miR-6866-5p, which encodes a different miRNA that targets GRID2 and GABRB2 [25], proposing new hypotheses regarding the putative roles of miRNAs that influence the GABAergic and glutamatergic pathways in smoking addiction. Otherwise, two SNPs in *CHRNA3* had statistically significant differences in genotype frequencies, rs1317286 and rs615470. Using a family-based association test, Li and colleagues found a nominal association rs1317286 and rs8040868 in *CHRNA3* with nicotine dependence in the African Americans and combined with European American samples. However, this association was not significant after correction for multiple testing [26]. Finally, the rs17408276 and rs951266 in the *CHRNA5* gene were found in different genotype frequencies among groups; the rs17408276 variant moderates nicotine deprivation, a neural index of cognitive control [27], and was associated with increased lung cancer risk in African Americans [28]. In comparison, rs951266 has been reported to be associated with schizophrenia and bipolar disorder [29].

This study is not exempt from limitations. The first one refers to the sampling method. In this study, only patients’ samples from the previously published e-Decídete! program were included, and maybe the most important is the reduced sample size compared to others; current results also only showed baseline data from two different interventions. Later studies including the risk of relapse after the end of treatment are desirable. In addition, no other potential confounders (as marital or socio-economic status) were recorded among the participants. Besides, although the Fagerström test is a suitable instrument to evaluate nicotine addiction, each consumer has their style for smoking (puff number, volume, interval, and duration); we were not able to analyze blood or urine cotinine levels in the on-site group to ascertain the self-reported cigarettes per day smoked by the subjects.

The main strength is the comparison of two different interventions for quitting smoking. Moreover, this is the first time a mobile-based smoking cessation intervention has been compared in terms of smokers’ genetic characteristics. This collaborative research establishes the basis for future investigations where smoking cessation interventions can be aborded from a genetic perspective. Among the practical implications of this study is the characterization of the smoking behavior of subjects attending two different smoking cessation interventions. Future research efforts should focus on fully characterizing the functional genetic variation and identifying the impact of these functional variants on smoking and treatment outcomes.

## 5. Conclusions

Tobacco smoking subjects attending a mobile smoking cessation intervention smoke fewer cigarettes per day by fewer years and fewer cumulative pack-years. There are differences in the genotype frequencies of SNPs in genes related to nicotine metabolism (*CYP2A6*) and nicotine dependence (*NRXN1*, *DRD4*, *CHRNA3*-*CHRNA5*). Slow metabolizers smoke more cigarettes per day than intermediate and normal metabolizers.

## Figures and Tables

**Table 1:** Molecular characteristics and frequencies for SNPs in *CYP2A6* related to the enzymatic activity.

SNP	Chr Position	Allele Change	MAF (GnomAD)	Consequence/Gene Location	Residue	
					Change	Position
rs28399433	chr19:40850474	A > C	C = 0.10323	2KB Upstream Variant	NA	NA
rs28399434	chr19:40850414	C > T	T = 0.00003	Missense Variant	G [Gly] ⇒ R [Arg]	5
rs8192720	chr19:40850405	G > A	A = 0.02214	Synonymous Variant	L [Leu] ⇒ L [Leu]	8
rs1137115	chr19:40850376	T > C	T = 0.24442	Synonymous Variant	V [Val] ⇒ V [Val]	17
rs1801272	chr19:40848628	A > T	T = 0.01974	Missense Variant	L [Leu] ⇒ H [His]	160
rs199916117	chr19:40848293	T > C	C = 0.00058	Missense Variant	K [Lys] ⇒ E [Glu]	194
rs148166815	chr19:40845404	A > G	G = 0.00008	Missense Variant	Y [Tyr] ⇒ H [His]	351
rs1809810	chr19:40844759	T > A	A = 0.01259	Missense Variant	Y [Tyr] ⇒ F [Phe]	392
rs2431413	chr19:40844073	A > G	G = 0.12103	Intron Variant	NA	NA
rs143731390	chr19:40843969	T > A	A = 0.1020	Missense Variant	N [Asn] ⇒ Y [Tyr]	438
rs5031016	chr19:40843869	A > G	G = 0.00775	Missense Variant	I [Ile] ⇒ T [Thr]	471
rs6413474	chr19:40843854	T > C	C = 0.01235	Missense Variant	K [Lys] ⇒ R [Arg]	476
rs5031017	chr19:40843845	C > A	A = 0.00049	Missense Variant	G [Gly] ⇒ V [Val]	479
rs28399468	chr19:40843827	C > A	A = 0.00165	Missense Variant	R [Arg] ⇒ P [Pro]	485

SNP: single nucleotide polymorphism; Chr: chromosome; MAF: minor allele frequency; GnomAD: genome aggregation database; NA: not apply.

**Table 2:** Characteristics of polymorphisms previously reported on the fine mapping of six genomic regions.

SNP	Gene	Chr Position	Allele Change	MAF (GnomAD)	Consequence/Gene Location
rs10189159	*NRXN1*	chr2:50487433	T > C	C = 0.3286	Intron Variant
rs985919		chr2:50459875	C > A	A = 0.4957	Intron Variant
rs10865246		chr2:50443116	C > A	C = 0.4950	Intron Variant
rs1882296		chr2:50039707	T > C	C = 0.3900	Intron Variant
rs1800955	*DRD4*	chr11:636784	T > C	C = 0.406	2KB Upstream Variant
rs6311	*HTR2A*	chr13:46897343	C > T	T = 0.3970	2KB Upstream Variant
rs6313		chr13:46895805	G > A	A = 0.40701	Synonymous Variant
rs12914385	*CHRNA3*	chr15:78606381	C > A	T = 0.3138	Intron Variant
rs1317286		chr15:78603787	A > G	G = 0.2974	Intron Variant
rs6495307		chr15:78597979	C > T	T = 0.310	Intron Variant
rs615470		chr15:78593646	T > C	T = 0.3688	Intron Variant
rs16969968	*CHRNA5*	chr15:78590583	G > A	A = 0.26553	Missense Variant
rs17408276		chr15:78589276	T > C	C = 0.2881	Intron Variant
rs951266		chr15:78586199	G > A	A = 0.2522	Intron Variant
rs680244		chr15:78578946	T > C	T = 0.4098	Intron Variant
rs17486278		chr15:78575140	A > C	C = 0.3167	Intron Variant
rs4105144	*CYP2A6* near	chr19:40852719	T > C	C = 0.4412	Intergene

SNP: single nucleotide polymorphism; Chr: chromosome; MAF: minor allele frequency; GnomAD: genome aggregation database; NA: not apply.

**Table 3:** Demographical variables.

Variable	e-Dec (*n* = 38)	On-Site (*n* = 94)	*p* *
Age (years)	35 (20–60)	36 (20–40)	0.700
Male/Female *n*, (%)	24/14 (63.2/36.8)	57/37 (60.6/39.4)	0.942
Cigarettes per day	9 (2–30)	10 (1–60)	0.064
Years of smoking	12 (2–44)	16 (1–43)	0.049
Onset smoking age	20.5 (12–50)	17.5 (9–50)	0.004
Tobacco index	6 (0.6–66.0)	9.8 (0.1–38.7)	0.015

Median (minimum and maximum values). * *p*-value with Mann-Whitney U test.

**Table 4:** Genotype and allele frequencies in both groups included in this study only in six SNPs of *CYP2A6*.

SNP Genotype/Allele	e-Dec		On-Site		*p* *
	*n* = 38	GF/AF %	*n* = 94	GF/AF %	
rs28399433					
TT	30	78.95	32	34.04	<0.0001
TG	7	18.42	59	62.77	
GG	1	2.63	3	3.19	
T	67	88.16	123	65.43	0.0003
G	9	11.84	65	34.57	
rs28399434					
GG	38	100.00	88	93.62	NS
GA	0	0.00	6	6.38	
AA	0	0.00	0	0	
G	76	100	182	96.81	NS
A	0	0	6	3.19	
rs8192720					
CC	37	97.37	94	100	NS
CT	1	2.63	0	0	
TT	0	0.00	0	0	
C	75	98.68	188	100.00	NS
T	1	1.32	0	0.00	
rs1137115					
GG	27	71.05	69	73.4	NS
GA	10	26.32	23	24.47	
AA	1	2.63	2	2.13	
G	64	84.21	161	85.64	NS
A	12	15.79	27	14.36	
rs2431413					
TT	38	100.00	69	73.4	NS
TC	0	0.00	22	23.4	
CC	0	0.00	3	3.19	
T	76	100.00	160	85.11	NS
C	0	0.00	28	14.89	
rs5031017					
GG	19	50.00	43	45.74	NS
GT	13	34.21	35	37.23	
TT	6	15.79	16	17.02	
G	51	67.11	121	64.36	NS
T	25	32.89	67	35.64	

* *p*-Pearson’s χ^2^. GF: genotype frequency, AF: allele frequency, NS: non-significant.

**Table 5:** Genotype and allele frequencies in both groups included in this study in *NRXN1*, *DRD4*, *HTR2A*, *CHRNA3*, *CHRNA5*, and near *CYP2A6*.

Gene/SNP Genotype/Allele	e-Dec		On-Site		*p* *
	*n* = 38	GF/AF %	*n* = 94	GF/AF %	
*NRXN1*					
rs10189159					
TT	32	84.21	44	46.8	0.0004
TC	5	13.16	45	47.87	
CC	1	2.63	5	5.32	
T	69	90.79	133	70.74	0.0009
C	7	9.21	55	29.26	
rs985919					
TT	21	55.26	29	30.85	0.0333
TG	14	36.84	54	57.44	
GG	3	7.89	11	11.7	
T	56	73.68	112	59.57	0.0437
G	20	26.32	76	40.43	
rs10865246					
AA	21	55.26	38	40.42	0.0621
AC	15	39.47	42	44.68	
CC	2	5.26	14	14.89	
A	57	75.00	118	62.77	0.0783
C	19	25.00	70	37.23	
rs1882296					
AA	26	68.42	45	47.87	0.0348
AG	11	28.95	43	45.74	
GG	1	2.63	6	6.38	
A	63	82.89	133	70.74	0.0589
G	13	17.11	55	29.26	
*DRD4*					
rs1800955					
TT	19	50	43	45.74	0.2936
TC	17	44.74	37	39.36	
CC	2	5.26	14	14.89	
T	55	72.37	123	65.43	<0.0001
C	21	27.63	65	34.57	
*HTR2A*					
rs6311					
GG	19	50	35	37.23	0.3103
GA	14	36.84	46	48.93	
AA	5	13.16	13	13.82	
G	52	68.42	116	61.70	0.3750
A	24	31.58	72	38.30	
rs6313					
CC	22	57.89	77	81.91	0.0772
CT	14	36.84	9	9.57	
TT	2	5.26	8	8.51	
C	58	76.32	163	86.70	0.0594
T	18	23.68	25	13.30	
*CHRNA3*					
rs12914385					
CC	23	60.53	49	52.12	0.6203
CT	12	31.58	40	42.55	
TT	3	7.89	5	5.32	
C	58	76.32	138	73.40	0.7380
T	18	23.68	50	26.60	
rs1317286					
AA	28	73.68	49	52.12	0.0311
AG	9	23.68	40	42.55	
GG	1	2.63	5	5.32	
A	63	82.89	138	73.40	0.1390
G	13	17.11	50	26.60	
rs6495307					
CC	29	76.32	53	56.38	0.0903
CT	7	18.42	37	39.36	
TT	2	5.26	4	4.25	
C	65	85.53	143	76.06	0.1240
T	11	14.47	45	23.94	
rs615470					
TT	31	81.58	57	60.63	0.0355
TC	6	15.79	33	35.1	
CC	1	2.63	4	4.25	
T	68	89.47	147	78.19	0.0499
C	8	10.53	41	21.81	
*CHRNA5*					
rs16969968					
GG	25	65.79	53	56.38	0.3700
GA	11	28.95	35	37.23	
AA	2	5.26	6	6.38	
G	61	80.26	141	75.00	0.4510
A	15	19.74	47	25.00	
rs17408276					
TT	31	81.58	53	56.38	0.0161
TC	6	15.79	39	41.48	
CC	1	2.63	2	2.12	
T	62	81.58	145	77.13	0.5280
C	14	18.42	43	22.87	
rs951266					
CC	36	94.74	51	54.25	<0.0001
CT	2	5.26	38	40.42	
TT	0	0	5	5.31	
C	74	97.37	140	74.47	<0.0001
T	2	2.63	48	25.53	
rs680244					
GG	27	71.05	52	55.32	0.1006
GA	10	26.32	37	39.36	
AA	1	2.63	5	5.31	
G	64	84.21	141	75.00	0.1430
A	12	15.79	47	25.00	
rs17486278					
AA	24	63.16	46	48.93	0.3213
AC	11	28.95	43	45.74	
CC	3	7.89	5	5.31	
A	59	77.63	153	81.38	0.6000
C	17	22.37	35	18.62	
near to *CYP2A6*					
rs4105144					
TT	26	68.42	62	65.95	0.9709
TC	10	26.32	29	30.85	
CC	2	5.26	3	3.19	
T	62	81.58	153	81.38	0.9701
C	14	18.42	35	18.62	

* *p*-Pearson’s χ^2^. GF: genotype frequency; AF: allele frequency.

## Data Availability

All data generated for this study are included in this article and its supplementary information file.
